# Coccidioidomycosis-Attributable Death in the United States: An Analysis of Cases Reported on Death Certificates, 2018–2023

**DOI:** 10.3390/jof11110766

**Published:** 2025-10-24

**Authors:** Huiqiao Fan, Fariba Donovan, Belinda Lovelace, Craig I. Coleman

**Affiliations:** 1 Department of Pharmacy Practice, School of Pharmacy, University of Connecticut, Storrs, CT 06269, USA; huiqiao.fan@uconn.edu; 2 The Valley Fever Center for Excellence, University of Arizona College of Medicine-Tucson, Tucson, AZ 85724, USA; faribadonovan@arizona.edu; 3 Health Economics and Outcomes Research, F2G Inc., Princeton, NJ 08540, USA; belinda.lovelace@f2g.com

**Keywords:** coccidioidomycosis, mortality, death certificates

## Abstract

Contemporary data on coccidioidomycosis death rates are sparse. Death certificate data for 2018–2023 from the US National Vital Statistics System were evaluated. Coccidioidomycosis deaths were identified using diagnosis codes B38.x listed anywhere on certificates. Deaths and age-adjusted mortality rates (AAMRs)/1,000,000 people, with 95% confidence intervals (CIs), were determined. We identified 1760 coccidioidomycosis-attributable deaths (AAMR = 0.75; 0.72–0.79). Most occurred in 55–74-year-olds (43.9%; corresponding AAMR = 1.72; 1.59–1.84). Males had a 2.69-fold increased AAMR versus females and American Indian or Alaska Native individuals had a 4.28-fold increased rate versus White individuals. Hispanics had a higher AAMR than the overall population (AAMR = 1.92; 1.76–2.08). AAMRs increased from 0.52 in 2019 to 0.79–0.94 in later years. Most (89.7%) death certificates were from endemic states, with Arizona having the highest AAMR. Seven hundred and thirteen certificates (40.5%) listed coccidioidomycosis as the primary cause of death, with 43.8% coded for pulmonary, 34.9% coded for disseminated, and 21.3% coded for unspecified coccidioidomycosis. Diabetes, COVID-19, and human immunodeficiency virus were more frequent on certificates with coccidioidomycosis versus without (RR range = 1.47–17.20). Mortality remained closely tied to demographic and geographic factors identified in prior studies, with county-level mapping revealing high-burden areas for targeted intervention. Coccidioidomycosis-attributable AAMRs rose over time, possibly influenced by concurrent COVID-19 infection. Only 40% of death certificates listed it as the primary cause, indicating that most patients experience chronic infection rather than death directly from the disease. These findings suggest the need for heightened clinical awareness of coccidioidomycosis, along with earlier diagnosis and prompt initiation of antifungal treatment in these high-risk groups.

## 1. Introduction

Coccidioidomycosis, also known as Valley fever, is a systemic fungal infection caused by the dimorphic *Coccidioides* species, which in the United States (US) is endemic to Central and Southern California, the deserts of Arizona, Southeastern New Mexico, and Western Texas, and various other areas in the southwest region [1,2]. Each year, approximately 20,000 cases of coccidioidomycosis are reported in the US [1,2,3,4]; however, the true estimated burden of coccidioidomycosis is thought to be at least 10-fold higher than the statistic reported to the Centers of Disease Control and Prevention (CDC), with estimates closer to 273,000 cases per year [5]. Moreover, updated estimates suggest that there are ~23,000 coccidioidomycosis hospitalizations and 900 deaths annually.

Antifungal therapy for coccidioidomycosis is determined by the location and severity of infection [1]. Coccidioidal infections are often treated with triazoles (e.g., fluconazole) or liposomal amphotericin B. Duration of therapy is variable, and if indicated, can range from 3–6 months for pulmonary disease to lifelong treatment in the cases of disseminated and meningeal coccidioidomycosis [1].

As new antifungal therapies with activity against coccidioidomycosis undergo clinical development [2,6], it is increasingly important to better understand the epidemiology of coccidioidomycosis and describe populations with the greatest unmet need and highest mortality rates.

Death certificate data from the National Vital Statistics System (NVSS) provide comprehensive national coverage of mortality in the US, capturing causes of death (using International Classification of Disease—Tenth Revision (ICD-10) coding), demographic, and geographic details [7,8]. Though not without limitations (i.e., sparse clinical detail and the potential for misclassification bias), death certificate data enable consistent estimation and comparison of age-adjusted mortality rates across populations and over time [7,8].

We sought to characterize US death certificates from 2018 to 2023 reporting coccidioidomycosis as a cause of death, whether as primary (underlying) or contributing, and calculate and compare contemporary death counts and mortality rates stratified by key characteristics. Additionally, we assessed the proportion of coccidioidomycosis versus non-coccidioidomycosis death certificates listing key comorbidities to identify factors potentially associated with coccidioidomycosis mortality.

## 2. Materials and Methods

We performed a retrospective observational cohort study to estimate mortality rates associated with coccidioidomycosis as a primary (underlying) or contributing cause of death. ‘Multiple cause of death’ data from the NVSS for 2018–2023 were analyzed using the CDC Wide-ranging ONline Data for Epidemiologic Research (WONDER) platform [7,8]. Cause of death data was drawn from death certificates for US residents collected by all 50 states and the District of Columbia. Each death certificate had a single primary cause of death, up to 20 contributing causes, and demographic (e.g., year of death, age, sex, race, ethnicity, geographic location) data. Causes of death listed on certificates were reported using four-character alphanumeric ICD-10 diagnosis codes. Death due to coccidioidomycosis was established based on the presence of an ICD-10 code of B38.x anywhere (primary or contributing position) on the death certificate.

The number of reported deaths and age-adjusted mortality rates (AAMRs) per 1,000,0000 people, along with 95% confidence intervals (CIs), were calculated using 2000 US Census Bureau standard estimates [9]. Age-adjusted mortality rates allow for comparisons of death rates between populations that have different age distributions. Number of reported deaths and corresponding AAMRs were stratified by underlying versus contributing cause of death, age (<55, 55–74, ≥75), year of death (2018–2023), sex, race (White, Black, Asian, American Indian or Alaska Native, or Other, ethnicity (Hispanic, non-Hispanic), and geographic location [Arizona, California, other endemic states (Nevada, New Mexico, Texas, Utah, Washington), non-endemic or unknown endemicity states]. Results of analysis of endemic states at the state and county levels were reported as crude mortality rates due to CDC reporting limitations. Mortality rates representing <10 deaths were suppressed, and <20 deaths were marked as unreliable per data reporting rules [8]. The underlying cause of death for all coccidioidomycosis-coded patients (using the CDC’s standard list of 113 selected categorized causes of death [7]) was reported as proportions. We calculated risk ratios (RRs) with 95% confidence intervals (CI) to compare relative AAMRs within each demographic, with the lowest rate category chosen as referent. To identify possible comorbidities associated with coccidioidomycosis-attributable death, the proportion of coccidioidomycosis death certificates with a specific comorbidity (i.e., chronic lung disease, COVID-19, diabetes, hematologic cancer, or human immunodeficiency virus (HIV) identified by ICD-10 codes) were compared with the number of non-coccidioidomycosis-attributable death certificates with the same comorbidity (all non-coccidioidomycosis death certificates were included in these analyses). RRs were also used to compare the relative reporting of comorbidities on coccidioidomycosis and non-coccidioidomycosis death certificates.

All analyses and visualization were performed in R version 4.4.1 (R Foundation for Statistical Computing, Vienna, Austria).

The data were accessed in compliance with the Health Insurance Portability and Accountability Act (HIPAA). Institutional review board approval was not required for this analysis of publicly available, de-identified secondary data.

## 3. Results

Between 2018 and 2023, 18,912,824 deaths were reported in the US. Of these, 34,824 were attributable to fungal infections, and 1760 (5.1% of all fungal) deaths had coccidioidomycosis listed anywhere on the death certificate. The overall AAMR for coccidioidomycosis over this time was 0.75 (95% CI: 0.72–0.79) deaths per 1,000,000 people.

### 3.1. Coccidioidomycosis Death by Demographic

The largest number of deaths occurred in patients between 55 and 74 years old (43.9%) (Table 1). More deaths occurred in males (69.3%; AAMR: 1.13, 95% CI: 1.06–1.19), a significant 2.69-fold increase compared to females. While most coccidioidomycosis-associated deaths occurred in Whites (76.0%), followed by Blacks (11.6%) and Asians (6.1%), all three races had similar AAMRs (range: 0.71–0.84). American Indian or Alaska Natives had a 4.28-fold increased rate versus Whites. Hispanics had a higher AAMR than the overall population (AAMR: 1.92; 95% CI: 1.76–2.08).

### 3.2. Death by Year

Between 192 and 366 coccidioidomycosis-attributable deaths occurred annually between 2018 and 2023. Compared to 2019, AAMRs were significantly increased in later years (RR increases ranging from 52–81%). The peak AAMR of 0.94 (0.84–1.04) occurred in 2021, with 366 deaths.

### 3.3. Geography

**Figure 1:**
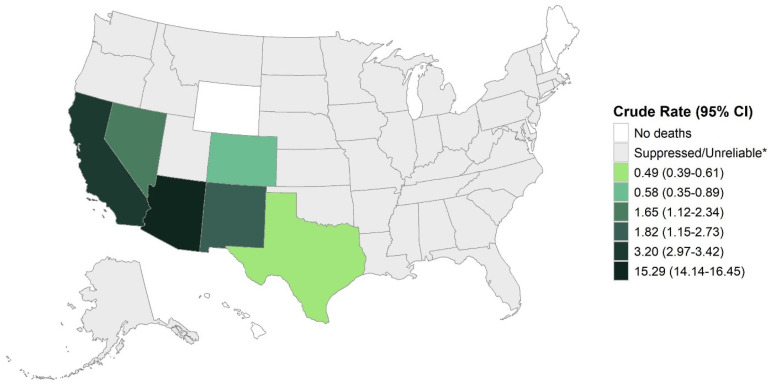
Crude Rates of Coccidioidomycosis-Attributable Death Per 1,000,000 People by State. CI = confidence interval. * Suppressed counties had <10 deaths and unreliable counties had <20 deaths.

**Figure 2:**
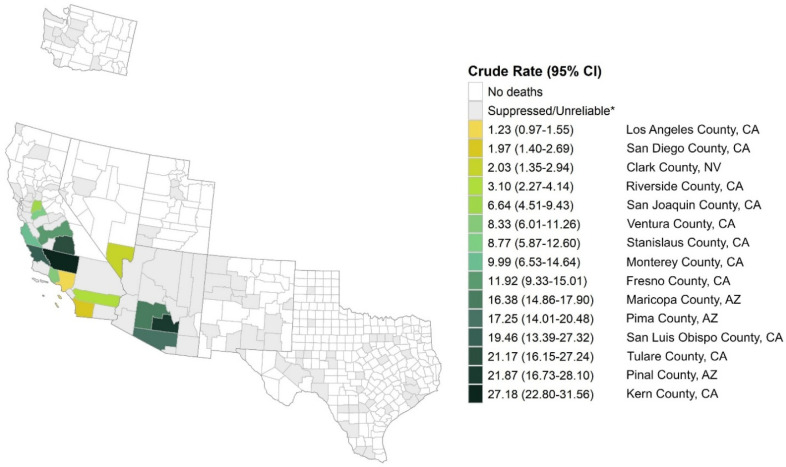
Crude Rate of Coccidioidomycosis Death Per 1,000,000 People by County Within Endemic States. AZ = Arizona; CA = California; CI = confidence interval; NV = Nevada. * Suppressed counties had <10 deaths. Unreliable estimate: counties had <20 deaths.

Of the 1760 coccidioidomycosis-attributable deaths, 1578 (89.7%) occurred in the six endemic states (Figure 1). Most deaths occurred in California (753/1760, 42.8%), followed by Arizona (672/1760, 38.2%), though Arizona had the highest AAMR (12.43; 95% CI: 11.46–13.40) of all states, representing 113.00 and 25.91-fold increases in mortality compared to non-endemic/unknown endemicity states. Low endemic states (New Mexico, Nevada, Texas, Utah, and Washington) had a greater number of deaths due to coccidioidomycosis than non-endemic/unknown endemicity states, resulting in a significantly higher (5.36-fold) mortality rate. Within endemic states, the rate of coccidioidomycosis-attributable deaths was not uniformly distributed across counties. Crude mortality rates per county within endemic states are depicted in Figure 2 and Table S1 and show that the highest AAMR was confined to relatively small areas within California, Arizona and Nevada.

### 3.4. Coccidioidomycosis Codes Utilized

**Figure 3:**
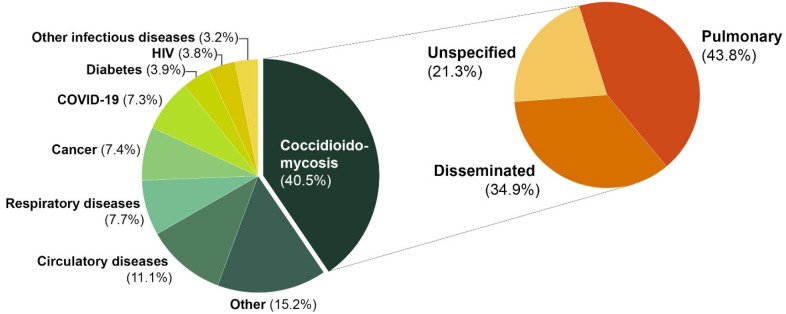
Underlying (Primary) Causes of Death on Certificates that Include Coccidioidomycosis (N = 1760). HIV = human immunodeficiency virus. Other infectious diseases included International Classification of Diseases, Tenth Revision codes for A00-B37, B39-B99; respiratory diseases (J00-J98), cancer (C00-D48), and circulatory diseases (I00-I99).

A plurality (713, 40.5%) of cases had coccidioidomycosis listed as the primary cause of death, of which, 43.8% were coded for pulmonary (ICD-10 B38.0-B38.2) (81.4% pulmonary, unspecified), 34.9% disseminated (ICD-10 B38.3, B38.4, B38.7) (12.3% meningeal), and 21.3% unspecified coccidioidomycosis (ICD-10 B38.9) (Figure 3). Amongst the coccidioidomycosis-attributable deaths, other diseases, circulatory, and respiratory diseases were the second, third and fourth highest causes listed.

When looking at coccidioidomycosis as a contributing cause of death (proportions not mutually exclusive and total to >100%), ICD-10 codes for pulmonary manifestations (acute, chronic, and/or unspecified) were the most often listed (45.7%), followed by ICD-10 codes for unspecified (29.8%), and disseminated (25.5%) including meningeal coccidioidomycosis (10.4%).

### 3.5. Comorbidities

Coccidioidomycosis death certificates listed chronic lung disease (10.9%), COVID-19 (9.1%), diabetes (18.4%), hematologic malignancy (3.0%) and HIV (4.2%) (Table 2) anywhere on the death certificates. Of these, diabetes, COVID-19 and HIV were found to be more prevalent on death certificates of those with coccidioidomycosis compared to those without, while hematologic malignancy (though the number of these deaths was small (N = 52) on coccidioidomycosis-attributable death certificates) and chronic lung disease were not more prevalent on death certificates of those with coccidioidomycosis. COVID-19 was also found to be more prevalent on death certificates of those with pulmonary coccidioidomycosis compared to those without (RR 1.61, 95% CI: 1.31–1.99).

## 4. Discussion

Between 2018 and 2023, we found 1760 reported deaths with coccidioidomycosis listed anywhere on the death certificate, corresponding to an AAMR of 0.75 (95% CI 0.72–0.79) per 1,000,000 people. The largest number of reported coccidioidomycosis-attributable deaths occurred in 2021 and in patients between 55 and 74 years old. Relative mortality rates were found to be elevated in people of male sex, American Indian or Alaska Natives, Hispanic ethnicity, living in Arizona and California, and to a lesser extent, people living in low-endemic states. Diabetes, HIV, and COVID-19 were more prevalent on death certificates of those with coccidioidomycosis compared to those without coccidioidomycosis. The identified association between these at-risk populations suggests the need for heightened awareness of coccidioidomycosis by clinicians and patients, along with earlier diagnosis and timely initiation of antifungal treatment in these high-risk groups. In regard to COVID-19, strategies for prevention of COVID-19 co-infection (e.g., vaccination, face masks) should be encouraged [10].

Nationwide and in high endemic states, our observed AAMRs were higher than those reported in a prior study of coccidioidomycosis published by Huang and colleagues (overall AAMR 0.59; 95% CI 0.57–0.6; AAMR for Arizona 10.60; 95% CI 9.94–11.25; AAMR for California 2.47; 95% CI 2.35–2.60), which used the same data source as our analysis but spanning an earlier time frame (1990–2008) [4]. Within our analysis, we also observed a pattern of increased AAMRs over time (0.52 in 2019 up to 0.79–0.94 in subsequent years) which were not reported by Huang et al. These observations may, in part, be explained by the COVID-19 pandemic. In a study by Gold and colleagues [11], AAMRs for all death certificates listing fungal infections were shown to have increased during the years 2020–2021 compared to previous years, with the increases attributed to COVID-19–associated deaths. Of note, we found COVID-19 was more likely to be listed as a comorbidity on death certificates in those dying with coccidioidomycosis compared to those who did not. This further suggests a possible association between COVID-19 and coccidioidomycosis death [10,11].

About 40% of certificates listed coccidioidomycosis as the primary (underlying) cause of death, suggesting that people often died with, and not necessarily directly due to, coccidioidomycosis. Circulatory diseases (including stroke), respiratory diseases (including COVID-19), and hematologic malignancy were among the most frequent alternative primary causes of death reported in our study. Notably, the CDC has identified these same disease states as the top causes of death in the general US population [12].

One unique aspect of our study was our analysis of coccidioidomycosis-attributable deaths by ICD-10 code diagnosis subtypes. For the primary cause of death, 43.8% were coded for pulmonary, 34.9% disseminated, of which 12.3% were meningeal, and 21.3% unspecified coccidioidomycosis. When looking at coccidioidomycosis as a contributing cause of death, pulmonary was the most common manifestation listed on death certificates, about one-quarter of deaths were coded as disseminated disease (including meningitis), and nearly 30% of deaths were coded as ‘unspecified’. In both analyses, a substantial proportion of cases utilized the ‘unspecified’ ICD-10 code on death certificates, and consequently, we were unable to determine specific coccidioidomycosis manifestation(s) for all patients. Per Centers for Medicare and Medicaid Services (CMS) and the National Center for Health Statistics (NCHS) guidance, codes titled ‘unspecified’ should be reserved for use when the information in the medical record is insufficient to assign a more specific code [13]. What proportion of these ‘unspecified’ coccidioidomycosis deaths truly lacked sufficient detail to warrant a more definitive code is unclear. Future research to gain a better understanding of what constitutes this ‘unspecified’ cohort is needed to enhance the utility of future epidemiologic studies of coccidioidomycosis.

An important contribution of our study is the reporting of coccidioidomycosis-attributable mortality at the county level. Even within high-endemic states such as Arizona and California [14], we observed substantial variation in mortality rates across counties. County-specific mortality data can be used to target public health interventions and support the efficient allocation of resources to high-risk areas, including those affected by health disparities such as environmental, racial, and socioeconomic factors and access to care [5,11,15]. Furthermore, county-level data can inform research and, when monitored over time, support the assessment of prevention and treatment strategies.

Recently, Williams and colleagues [5] published a cross-sectional modeling study that extrapolated reported case counts of coccidioidomycosis to account for underreporting, underdiagnosis, and symptomatic case patients who did not seek healthcare. Their study suggested substantial under-reporting of coccidioidomycosis diagnosis and associated mortality in the US, with 2019 modeled “true” diagnosis estimates being 10–18 times higher than those “reported” by the National Notifiable Diseases Surveillance System (NNDSS) and 2019 coccidioidomycosis-attributable mortality counts 5–6-fold higher than that “reported” by the NVSS. Interestingly, our study’s estimate of ~300 deaths/year, while less than that of Williams et al., was still nearly twice as high as the 160 deaths/year figure commonly referred to in the medical literature [1]. As our study used “reported” mortality data, our AAMRs may represent an under-reporting of deaths compared to the “true” modeled values. If we applied the 5–6-fold higher mortality multiplier to our underlying coccidioidomycosis cause of death data (~119 deaths/year), our observed rate would be similar to what the model predicts. However, Williams et al.’s study did not suggest varying degrees of underreporting of mortality in specific demographics or by state or county. Thus, it is reasonable to assume the RRs reported in our study would be similar regardless of whether reported versus modeled estimates were utilized.

There are limitations that need to be considered when interpreting the results of this study. First, there is concern for misclassification bias due to our dependence on billing codes to identify coccidioidomycosis-attributable deaths (not laboratory-confirmed cases) [16]. At present, there are no published coding validation studies for coccidioidomycosis as a whole or any of its individual manifestations. Second, the results depicted in our study represent stratifications of one characteristic at a time. Consequently, the results of stratified analyses must be viewed against the risk of confounding. For example, while males were shown (in our and prior studies) to be associated with higher AAMRs than females, the increased risk may be due to males having a higher risk of exposure to coccidioidomycosis due to more frequent work in high-exposure occupations, such as construction, as well as underlying biological causes such as hormonal differences [17,18]. Third, counties that were less densely populated may have had substantial AAMRs that were non-calculable due to a small (<20) absolute number of deaths. Finally, prior studies have shown that errors on death certificates are not uncommon, with anywhere from 17 to 34% of certificates requiring some alteration to the cause or manner of death [19,20]. The need for these changes has been hypothesized to be a result of physicians’ unfamiliarity with the deceased, premature determination of the cause of death, time constraints in completing certificates, and their perceived unimportance. Incorrect death certificates could result in the under-representation of coccidioidomycosis deaths in NVSS [21].

## 5. Conclusions

Coccidioidomycosis accounted for ~5% of all fungal infection-related death certificates. Diabetes, COVID-19, and HIV were the most frequent comorbidities associated with higher AAMRs. Mortality remained strongly linked to demographic and geographic factors identified in prior studies [4]. County-level mortality mapping identified high-burden areas where targeted treatment and prevention efforts may be warranted. Coccidioidomycosis-attributable AAMRs increased over time, possibly reflecting the impact of concurrent COVID-19 infection [10]. Only 40% of death certificates listing coccidioidomycosis identified it as the primary cause, with other causes largely representing leading U.S. mortality categories, suggesting that most affected patients live with chronic infection requiring ongoing antifungal therapy rather than dying directly from it. These findings refine the epidemiologic understanding of coccidioidomycosis and support geographically focused, risk-based public health interventions.

## Figures and Tables

**Table 1:** Numbers, Age-Adjusted Mortality Rates and Rate Ratios of Death Stratified by Key Characteristics.

Characteristic	Deaths (% of Total)	AAMR (95% CI)	RR (95% CI)
**Total**	1760 (100)	0.75 (0.72–0.79)	--
**Year of death**			
2018	253 (14.4)	0.68 (0.59–0.76)	1.31 (0.90–1.90)
2019	192 (10.9)	0.52 (0.45–0.60)	Referent
2020	319 (18.1)	0.79 (0.70–0.88)	1.52 (1.06–2.18)
2021	366 (20.8)	0.94 (0.84–1.04)	1.81 (1.27–2.57)
2022	316 (18.0)	0.81 (0.72–0.90)	1.56 (1.09–2.23)
2023	314 (17.8)	0.81 (0.72–0.90)	1.56 (1.09–2.23)
Age *			
<55 years old	503 (28.6)	0.36 (0.33–0.39)	Referent
55–74 years old	773 (43.9)	1.72 (1.59–1.84)	4.78 (3.84–5.94)
≥75 years old	484 (27.5)	3.50 (3.19–3.81)	9.72 (7.66–12.35)
**Sex**			
Male	1220 (69.3)	1.13 (1.06–1.19)	2.69 (2.20–3.29)
Female	540 (30.7)	0.42 (0.38–0.45)	Referent
**Race**			
White	1338 (76.0)	0.71 (0.67–0.75)	Referent
Black	204 (11.6)	0.73 (0.62–0.83)	1.03 (0.76–1.40)
Asian	108 (6.1)	0.84 (0.68–1.00)	1.18 (0.80–1.75)
American Indian or Alaska Native	72 (4.1)	3.04 (2.36–3.86)	4.28 (3.32–5.50)
Other ^#^	38 (2.2)	0.89 (0.61–1.25)	1.25 (0.87–1.80)
**Ethnicity**			
Hispanic ^†^	571 (32.4)	1.92 (1.76–2.08)	--
**States**			
AZ	672 (38.2)	12.43 (11.46–13.40)	113.00 (76.38–167.17)
CA	753 (42.8)	2.85 (2.64–3.06)	25.91 (17.57–38.20)
Low endemic (NV, NM, TX, UT, WA)	167 (9.5)	0.59 (0.50–0.68)	5.36 (3.35–8.58)
Other	168 (9.5)	0.11 (0.09–0.13)	Referent

AAMR = Age-adjusted mortality rate; CI = confidence interval; NM = New Mexico; NV = Nevada; RR = rate ratio; TX = Texas; UT = Utah; WA = Washington. * Reported as crude mortality rates. ^†^ Due to reporting restrictions regarding publishing statistics representing nine or fewer deaths, only data for the Hispanic cohort is presented. ^#^ Other races included Native Hawaiian or Other Pacific Islander, or more than one race.

**Table 2:** Prevalence and Ratios of the Proportion of Certificates for Coccidioidomycosis and Non-Coccidioidomycosis Deaths Listing Key Comorbidities.

Comorbidity	Prevalence on Coccidioidomycosis Death Certificates (%) N = 1760	RR (95% CI) vs. Non-Coccidioidomycosis Death Certificates ^†^
Diabetes	324 (18.4%)	1.69 (1.54–1.87)
COVID-19	160 (9.1)	1.47 (1.27–1.71)
HIV	74 (4.2)	17.20 (13.72–21.45)
Hematologic malignancy	52 (3.0)	1.26 (0.96–1.64)
Chronic lung disease *	192 (10.9)	1.02 (0.89–1.16)

CI = confidence interval; HIV = Human immunodeficiency virus, RR = risk ratios. * Chronic lung disease = asthma, chronic obstructive pulmonary disease, and bronchiectasis. ^†^ N = 18,912,824 deaths without coccidioidomycosis listed.

## Data Availability

The original data presented in the study are openly available at the National Center for Health Statistics, Vital Statistics Online Data Portal, https://www.cdc.gov/nchs/data_access/vitalstatsonline.htm (accessed on 1 Februray 2025).

## Supplementary Materials

The following supporting information can be downloaded at https://www.mdpi.com/article/10.3390/jof11110766/s1, Table S1: Number of deaths, population size and crude rate of coccidioidomycosis death per 1,000,000 people by county within endemic states with at least 20 cases.
