# Supplementary material for: Coccidioidomycosis-Attributable Death in the United States: An Analysis of Cases Reported on Death Certificates, 2018–2023
Source: J Fungi (Basel). 2025 Oct 24;11(11):766. doi: 10.3390/jof11110766 (PMC12653847; doi:10.3390/jof11110766)
Supplement: Supplementary file 1 [file jof-11-00766-s001.zip › jof-3919510-supplementary.pdf]

SUPPLEMENTAL MATERIALS

Table S1. Number of Deaths, population Size and Crude Rate of Coccidioidomycosis Death Per 1,000,000 People by County Within Endemic States with At Least 20 Cases

County	Deaths	Population	Crude Rate Per 1,000,000
Kern County, CA (06029)	148	5,445,929	27.18<br>(22.80 - 31.55)
Pinal County, AZ (04021)	61	2,788,705	21.87<br>(16.73 - 28.10)
Tulare County, CA (06107)	60	2,834,802	21.17<br>(16.15 - 27.24)
San Luis Obispo County, CA (06079)	33	1,696,181	19.46<br>(13.39 - 27.32)
Pima County, AZ (04019)	109	6,320,316	17.25<br>(14.01 - 20.48)
Maricopa County, AZ (04013)	444	27,109,302	16.38<br>(14.85 - 17.90)
Fresno County, CA (06019)	72	6,040,352	11.92<br>(9.33 - 15.01)
Monterey County, CA (06053)	26	2,601,467	9.99<br>(6.53 - 14.64)
Stanislaus County, CA (06099)	29	3,306,260	8.77<br>(5.87 - 12.60)
Ventura County, CA (06111)	42	5,040,339	8.33<br>(6.01 - 11.26)
San Joaquin County, CA (06077)	31	4,666,379	6.64<br>(4.51 - 9.43)
Riverside County, CA (06065)	46	14,835,231	3.10<br>(2.27 - 4.14)
Clark County, NV (32003)	28	13,766,359	2.03<br>(1.35 - 2.94)
San Diego County, CA (06073)	39	19,846,371	1.97<br>(1.40 - 2.69)
Los Angeles County, CA (06037)	73	59,301,698	1.23<br>(0.96 - 1.55)

AZ=Arizona; CA=California; CI=confidence interval; NV=Nevada
